# Relationships Between Mobile eHealth Literacy, Diabetes Self-care, and Glycemic Outcomes in Taiwanese Patients With Type 2 Diabetes: Cross-sectional Study

**DOI:** 10.2196/18404

**Published:** 2021-02-05

**Authors:** Sophie Huey-Ming Guo, Hung-Chun Hsing, Jiun-Lu Lin, Chun-Chuan Lee

**Affiliations:** 1 Department of Nursing Mackay Medical College New Taipei City Taiwan; 2 Department of Nursing Hsinchu Cathay General Hospital Hsinchu Taiwan; 3 Division of Endocrinology and Metabolism Mackay Memorial Hospital Taipei Taiwan

**Keywords:** mHealth literacy, eHealth literacy, diabetes mellitus, self-care behavior, glycemic outcomes

## Abstract

**Background:**

Understanding how people with diabetes seek online health information and use health applications is important to ensure these electronic tools are successfully supporting patient self-care. Furthermore, identifying the relationship between patient mobile eHealth literacy (mobile eHL) and diabetes outcomes can have far-reaching utility, for example, in the design of targeted interventions to address mobile eHL limitations. However, only limited studies have explored the impact of mobile eHL in a population with diabetes.

**Objective:**

This study aims to present data about online information-seeking behavior and mobile health (mHealth) app usage, investigate the factors related to mobile eHL in Taiwanese patients with type 2 diabetes, and flesh out the relationship between eHealth literacy (eHL), mobile health literacy (mHL), and health outcomes.

**Methods:**

Subjects were recruited from January 2017 to December 2017 in the outpatient departments of 3 hospitals in Taiwan. A total of 249 Taiwanese patients with diabetes voluntarily completed a cross-sectional survey assessing sociodemographic characteristics; diabetes status; knowledge and skills of computers, the internet, and mobile apps; mobile eHL; and patient outcomes (self-care behaviors, self-rated health, HbA_1c_). Structural equation modeling analyses examined the model fit of mobile eHL scores and the interrelationships between latent constructs and observable variables.

**Results:**

Of the 249 patients with diabetes, 67% (164/249) reported they had searched for online diabetes information. The participants with smartphones had owned them for an average of 6.5 years and used them for an average of 4.5 (SD 3.81) hours per day. Only 1.6% (4/249) of the patients used health apps. Some demographic factors affecting mobile eHL included age, education, and duration of type 2 diabetes. Mobile eHL was related to self-care behaviors as well as knowledge and skills of computers, the internet, and mobile technology, but only had a weak, indirect effect on self-rated health. The final model had adequate goodness-of-fit indexes: chi-square (83)=149.572, *P*<.001; comparative fit index (CFI)=0.925; root mean square of approximation (RMSEA)=0.057 (90% CI 004-006); chi-square/*df*=1.082. Mobile eHL had a weak, indirect effect on self-rated health through the variables of knowledge with skills.

**Conclusions:**

Our study reveals that although people with diabetes who rated their health conditions as moderate were confident in using mobile eHealth and technology, few adopted these tools in their daily lives. The study found that mobile eHL had a direct effect on self-care behavior as well as knowledge and skills of computers, the internet, and mobile technology, and had an indirect effect on health outcomes (glycemic control and self-rated health status). Information about this population's experiences and the role mobile eHL plays in them can spur necessary mobile eHealth patient education.

## Introduction

### Diabetic Population in the Digital Age

Living with diabetes is a stressful condition that requires a great deal of self-care practices. By 2035, more than 1 in 10 adults worldwide will be at risk for developing diabetes [1]. With such a life-altering disease, this population must continuously obtain and process health information to cope. Today, a popular option for disseminating health information is via information technology, on websites accessed from mobile devices and personal computers [2]. In 2019, the rates for individual internet use and mobile internet use were 88% and 85.2%, respectively, according to the Taiwan Network Information Center [3]. The question arises as to whether Taiwanese people with diabetes turn to an online environment for health information or to engage with mobile health (mHealth) apps. Thus, understanding this issue is crucially important, as scholars point out that eHealth and mHealth tools can successfully support patients' self-care [4-7].

### eHealth and mHealth Technology

Recent advances in information technology can enhance patient self-care and optimize patient outcomes. One such advancement is eHealth, defined as the delivery of health services and information through electronic technologies [8]. Several web portals have been extensively developed for similar purposes, primarily to optimize patient outcomes [4,6]. Previous reviews have summarized the effectiveness of eHealth programs in regard to better self-management behaviors, adherence to medications and specific dietary recommendations, increased physical activity [4], and cost-effectiveness [9]. Clearly, there is great potential for the utility of eHealth technology to promote patient outcomes.

Mobile technology, an important addition to eHealth, also has great potential to support patient self-care. mHealth is characterized by a focus on mobile telecommunications for a variety of health care services and the implementation of smartphone applications for health purposes [10-12]. The literature shows that mHealth technology could be used to record medication, glycemic monitoring, and healthy food intake to optimize glycemic control [ie, reduction of glycated hemoglobin (HbA_1c_)] [7,13], and ultimately, to prevent diabetic complications [13].

The term mobile eHealth may comprehensively and simultaneously refer to both mHealth and eHealth [14,15], which have more potential together than either technology separately in the future provision of continuing care. Thus, some researchers have made efforts to increase the evidence base for mobile eHealth design and development [16,17]. Unfortunately, these valuable resources can only be helpful if patients have adequate access to them. Research indicates that some barriers still exist, such as limited user engagement [18-20] and technological issues [12,13]. One study [21], reviewing 101 apps, found that most health apps do not adhere to the guideline of mHealth-literate design strategies published by the Institute of Medicine. Due to these issues, patients have not widely embraced mHealth apps, despite their great promise.

### eHealth and mHealth Literacy

To overcome these adoption barriers, eHealth and mHealth literacy should be recognized and addressed when planning for these innovations. eHealth literacy can be viewed as an extension of health literacy, the ability to obtain, process, and understand health information [22]. Although the concept of eHealth literacy (eHL) almost resembles health literacy, eHL emphasizes electronic health information rather than traditional information sources (such as pamphlets and printed patient handouts). Finding relevant information online and judging its credibility goes beyond traditional health literacy measures [22,23]. Thus, eHL reflects the complexity inherent in the utility of information technologies for health. These skills are better captured by an eHealth literacy scale [22,24-26].

Another relatively new literacy, mHealth literacy (mHL), is generally viewed as the ability to adopt mobile devices to search, find, understand, appraise, and apply health information when addressing or solving health problems [27]. While the broad definition of mHL seems rather similar to the concept of eHL, the assessment of eHealth literacy cannot fully represent the mHL dimension. To be more precise, eHL does not include the ability to access mHealth apps, download from app stores, and register these apps. In the mHealth field, mHealth literacy is supposed to serve a literacy function, enabling people to properly operate mHealth apps. Unfortunately, the current mHL scale only emphasizes health information seeking and health information appraisal on mobile devices. Thus, this study proposes a mobile eHealth literacy scale by adding a new subscale of mHL items that was modified from the eHL scale of Norman and Skinner [22].

One fundamental challenge facing both eHL and mHL (mobile eHL) is the need to discover how a diverse population could use mobile eHealth technology to acquire health information. Patients with diabetes with lower levels of mobile eHL may not understand or be able to access electronic health information and mobile health apps [28]. To date, research into the combined measurement of eHL and mHL is scant. This lack of data is of great concern to health care providers of vulnerable patients, such as those with diabetes, who should seek reliable information and useful self-care tools [2,15,20,23].

### Relationship Between Mobile eHealth Literacy and Patient Outcomes

There is now a substantial body of literature that examines health literacy effects on disease conditions. Researchers have noted a causal mechanism associating health literacy to health outcomes (ie, utilization of health services, patient-provider interaction, and self-care) [29]. They have identified important pathways and highlighted the complexity involved in patient self-care. Other studies [30,31] demonstrate a logical, linked structure of health literacy, patient characteristics, self-care, and health outcomes. Scholars [31] indicated low to insufficient evidence on the association between health literacy, eHealth literacy, and self-rated health. Similarly, two other systematic reviews offer a comprehensive set of variables to ascertain health literacy skills, patient characteristics, and risk factors associated with clinical outcomes. In sum, these fundamental studies provide a preliminary understanding of health literacy based on an analysis of the literature, but they lack empirical confirmation.

Subsequent studies have sought to confirm these models with empirical findings. For example, one cross-sectional study validated a model that describes how health literacy contributes to physical activity and self-rated health among patients with chronic conditions [32]. Other research reveals that high health literacy has an indirect positive effect that can indirectly facilitate diabetes self-care and improve glycemic control [33]. In brief, health literacy positively relates to health outcomes in chronic diseases.

Evidence of traditional health literacy's capacity to regulate behaviors has been well established, but such evidence in the mobile eHealth field is still limited. As stated by Kim and Xie [23], there is a lack of new health literacy screening tools to identify proper competence in the use of eHealth and mHealth services. A mediation analysis by Schulz et al [25] found that eHealth literacy is weakly associated with health system utilization, though the premise of health literacy resembles eHealth literacy. Additionally, low eHealth literacy is associated with poor quality-of-life outcomes in patients with chronic lung disease [34]. Another research group found that mHealth apps users with heart disease or diabetes have a higher level of eHL [35]. These studies offer valuable insights about eHL but neglect to focus on mobile health literacy.

To sum up, health literacy has not yet been shown to accurately serve the needs of patients with diabetes who rely heavily upon the internet and mobile technology. An eHL measure [22,34] merely reflects internet use without specifically covering mobile apps. Also, the eHL measure has been questioned because it only reflects individuals' perceived performance of online tasks without necessary objective reports [31,36]. Researchers recommend that future studies measure people's internet operation skills in a manner that accurately reflects their eHL [6,36,37]. For this study, self-developed mHL questions and eHL items will be combined to flesh out the scope of mobile eHealth literacy; cross-verification will then be performed by an assessment of knowledge and skills of computer, mobile, and internet competence.

This study is one of the few to date that explores the link between mobile eHL and diabetes outcomes (ie, self-care behavior and glycemic control). As various mobile eHealth programs are particularly attractive for managing chronic conditions [6,13,16,18,38], there are far-reaching benefits to recognizing the relationship between patient literacy and chronic disease outcomes. Thus, identifying the factors linking eHL to behavioral and clinical outcomes in diabetes can contribute to future design projects.

### Objective

This study aims to present data about online information-seeking behavior and mHealth apps usage, to analyze the levels of eHealth and mHealth literacy of patients with diabetes, and to flesh out the relationship between eHL, mHL, and health outcomes (self-care behaviors, self-rated health, and HbA_1c_).

The following 4 hypotheses are examined: Hypothesis 1 posits that higher mobile eHL is associated with (1) greater knowledge and skills of computers, the internet, and mobile technology; (2) increased self-care behaviors; (3) better self-rated health; and (4) lower HbA_1c_. Hypothesis 2 posits that higher knowledge and skills of computers, the internet, and mobile technology is associated with (1) increased self-care behaviors, (2) better self-rated health, (3) and lower HbA_1c_. Hypothesis 3 posits that increased self-care behaviors are associated with lower HbA_1c_, and hypothesis 4 posits that better self-rated health is associated with lower HbA_1c_.

## Methods

### Recruitment and Participants

The study employed a cross-sectional survey. A self-administered questionnaire was completed during a 12-month period, from January 16, 2017, to December 15, 2017. Potential participants were referred by endocrinologists or certificated diabetes educators at 3 hospitals in Taiwan. The inclusion criteria included (1) ages 20-65 years, (2) basic reading and writing ability, (3) no vision defects, and (4) a willingness to participate in the research study. Subjects were excluded if they had severe vision loss, communication problems, or if they had alcohol or drug abuse issues.

From the outpatient department of endocrinology and metabolism, 262 patients were recruited. Eligible patients were interviewed in the outpatient department waiting rooms, which are safe, private, and secure. Our researchers introduced the procedure to each participant, including the study's purpose, the method, the time required to complete the questionnaire, and how the data would be used after it was collected. The patients were then administered the questionnaires and skill performance testing and received a gift card worth NT50 (USD $1.50) as an incentive for completing the survey.

### Ethics

This study was approved by the institutional review board of the targeted hospitals (IRB# 17MMHIS003e and CGH-OP105003) and was conducted in accordance with CONSORT-EHEALTH (Consolidated Standards of Reporting Trials of Electronic and Mobile Health Applications and Online TeleHealth) guidelines (Multimedia Appendix 1) [39]. All respondents were informed that participation was voluntary and that they could leave at any time without reason, and their choice to participate would not affect their care. All who chose to participate gave written consent.

### Measurements

#### Demographics

Demographic items included age, gender, education, health status, duration of diabetes, experience with mobile and internet use, and online health information-seeking habits. Additional measures included validated mobile eHealth literacy and knowledge and skills of mobile app and internet use. The health outcomes as dependent variables included self-rated health, diabetes self-care behavior, and HbA_1c_.

#### Mobile eHealth Literacy Questionnaire

The Mobile eHealth Literacy Questionnaire (Multimedia Appendix 2) consisted of 3 parts: (1) an existing scale [22] and self-developed measures in terms of eHealth literacy (8 items), (2) mHealth literacy (8 items), and (3) mobile eHealth preference (4 items). Firstly, eHealth literacy was examined using Norman and Skinner's eHEALS (eHealth Literacy Scale) [22] to measure perceived skills and comfort with using the internet for health information and decision-making. Factorial validity and internal consistency (Cronbach alpha=.94) were reported.

Secondly, our research group used available literature [10,15] to self-develop the mHealth literacy questionnaire by modifying Norman and Skinner's eHEALS [22]. Mobile skills were incorporated into the questionnaire to comprehensively measure all aspects of using internet resources through mobile technology [12]. This part asks questions about perceived skills concerning mobile health apps for self-management. Each item in the 2 subscales is rated on a 5-point Likert scale, in which 1=*strongly disagree* and 5=*strongly agree*. Higher scores indicate higher mHealth literacy.

Thirdly, the mobile eHealth preference was modified from eHEALS [22] and asked each individual their opinion on mHealth and eHealth technology. An example item is “How important is it for you to be able to access health resources on the internet?”

The content validity index for the 3 questionnaire subscales was completed by 6 senior experts (2 metabolism physicians, 2 dietitians, and 2 professors of health informatics) from 3 hospitals and 2 universities in Taiwan. The relevance, clarity, and simplicity of each item were evaluated using the content validity index. All individual items were rated above 3.5 on a 4-point scale. An item-level score of 3 or 4 indicated acceptable content validity [40]. Face validity was carried out with 3 voluntary participants with diabetes. Cronbach alpha for eHL, mHL, and mobile eHL preference scores were .927, .927, and .847, respectively.

#### Knowledge and Skills of Mobile Technology and the Internet

The knowledge and skills questionnaires examined the use of computers, the internet, and mobile apps, modified from Xie’s study [37]. This questionnaire was designed to offset the disadvantage of the eHL measure, which only reflects people's perceived performance on online tasks and lacks objective measures [36]. The knowledge-related test had 15 items that were each given a score of 1 if answered correctly or 0 if answered incorrectly. An example item is, “Try to find a pictogram meaning a place for downloading apps.”

The second skills-related test has 10 items. Each item scored 1 if operated appropriately and 0 if operated inappropriately; for instance, “Please try to open a browser and connect to a health website” and “Please try to download and use a diabetes app on a mobile device.” The Kuder-Richardson Formula 20 (KR-20) reliability for the knowledge and skills tests was 0.928 and 0.923, respectively. Face validity was carried out with 3 voluntary participants with diabetes.

#### Self-rated Health

Self-rated health derived by Hornby-Turner et al [41] was measured by asking subjects to respond to 3 questions; for example, “How would you describe your general health?” The reply scores ranged from 1=*very good* to 3=*poor*. Higher scores meant better-perceived health.

#### Diabetes Self-care Behavior Questionnaire

The 36-item Diabetes Self-care Behaviors questionnaire developed by Parchman et al [42] assesses the degree to which patients follow recommended self-care activities. For example, subjects are asked how frequently they comply with the recommended daily diet in a typical week. Behavior is measured on a 5-point ordinal scale: 0=*never*, 1=*1-3 times per week*, 2=*4-5 times per week*, 3=*more than 5 times per week*, 4=*always*. A higher score indicates more frequent self-care behavior.

#### Glycated Hemoglobin (HbA_1c_)

Glycated hemoglobin (HbA_1c_) is a critical index of glycemic control with the ability to reflect average blood glucose over a period of 3 months. The HbA1c data of the study subjects were collected by reviewing the electronic medical records in the enrollment period. The optimal range for HbA_1c_ is below 7.0 mg/dL. A higher level of HbA_1c_ indicates poor glycemic control, which is associated with a higher risk of vascular complications and death [43].

### Data Analysis

Data were analyzed using the SPSS statistical software package (version 22.0; IBM Corp). Data analysis included descriptive and exploratory statistical analyses. Pearson correlations evaluated the relations between independent variables and dependent scores. A structural equation model (SEM) was conducted to test the structure of the proposed model and the interrelationships between latent constructs and observable variables.

## Results

### Response Rate and Descriptive Statistics

Table 1 presents the demographic characteristics of the patients. Of the 262 participants, 23 were excluded due to incomplete data, and 249 were eligible for the final analysis. The mean age was 44.58 (SD 11.02; range 20-65) years. Of the 249 participants, 164 (65.9%) were men and 85 (34.1%) were women, and 60% (150/249) had an education level of at least college or university. The mean duration of type 2 diabetes was 6.14 (SD 5.6; range 0-26) years, and diabetes duration ranged from 1 to 5 years for 45.7% (112/249) of participants. Of the 249 subjects, 45.8% (115/249) reported their health status was fair. The average HbA_1c_ result was 7.96 (SD 1.89; range 5.3-15.2) mg/dL, and was over 7 mg/dL for 63.1% (157/249) of subjects.

**Table 1:** Sociodemographic characteristics of the study participants (n=249).

Characteristics		Values
**Gender, n (%)**		
	Male	164 (65.9)
	Female	85 (34.1)
Age in years, mean (SD; range)		44.58 (11.02; 20-65)
**Education level^a^ , n (%)**		
	High school or less	98 (39.5)
	College or university	114 (45.9)
	Master or PhD	36 (14.5)
**Duration of type 2 diabetes, mean (SD; range)^a^**		6.14 (5.60; 0-26)
	<1 year, n (%)	29 (11.8)
	1-5years, n (%)	112 (45.7)
	6-10 years, n (%)	59 (24.1)
	>10 years, n (%)	45 (18.3)
**Self-rated health, n (%)**		
	Good	115 (46.2)
	Fair	114 (45.8)
	Poor	20 (8)
**HbA_1c_ (mg/dL), mean (SD; range)**		7.96 (1.89; 5.3-15.2)
	<7, n (%)	92 (36.9)
	≥7, n (%)	157 (63.1)
	7.0-8.0, n (%)	72 (28.9)
	8.1-9.0, n (%)	31 (12.4)
	9.1-10.0, n (%)	20 (8.0)
	10.1-15, n (%)	34 (13.7)

^a^Some participant values are missing.

### The Use of eHealth, mHealth Technology, and Health Outcomes

Table 2 presents descriptive statistics. The participants were asked about their experience with health information technology, including diabetes information-seeking through the internet and the use of smart devices. Of the 249 participants, 68% (164/249) reported they had searched for online diabetes information. When asked about the information technology tools they owned, 239 of the 249 participants reported owning smartphones, 171 owned computers, and 89 owned a tablet. Regarding their daily usage of smart devices and health applications over the past 30 days, the participants with smartphones (owned, on average, for 6.5 years) used them for a daily average of 4.5 (SD 3.8) hours, while participants with tablet used them for a daily average of 2.2 (SD 2.6) hours. Only 1.6% (4/249) of respondents used health applications.

**Table 2:** Participant habits regarding online health information seeking and the use of mHealth apps (n=249).

Characteristics		Values
**Search for diabetes information, n (%)**		
	Have	164 (67.1)
	Have not	82 (32.9)
**Search for health information (n=134), n (%)**		
	Have	24 (17.9)
	Have not	110 (82.1)
**Use of health apps, n (%)**		
	Use (running app, DM app)	4 (1.6)
	Do not use	245 (98.4)
**Use of technology in years, mean (SD; range)**		
	Smartphone (n=239)	6.5 (3.3; 0-20)
	Tablet (n=89)	4.9 (3.2; 0-15)
	Computer (n=171)	15.9 (6.6; 0-40)
**Daily use in hours, mean (SD; range)**		
	Smartphone (n=239)	4.5 (3.8; 0-20)
	Tablet (n=78)	2.2 (2.6; 0-12)
	Computer (n=171)	4.8 (3.6; 0-20)

Table 3 presents the means, standard deviations, and Cronbach alpha values for self-rated health, self-care behavior, eHL, mHL, mobile eHL preference, and knowledge and skills of computers, internet, and mobile technology. Most measurements demonstrated good reliability (>0.8), and self-rated health showed moderate reliability (0.546). The overall eHL score averaged 30.16 (SD 5.41) on a scale of 8 to 40, the mHL score averaged 28.86 (SD 6.27) on a scale of 8 to 40, and the mobile eHL preference mean was 14.65 (SD 2.57) on a scale of 4 to 20. The average self-rated health score was 6.70 (SD 1.77), and the average self-care behavior score was 79.30 (SD 26.05).

**Table 3:** Means, standard deviations, and Cronbach alpha values for self-rated health, self-care behavior, eHealth literacy (eHL), mobile health literacy (mHL), mobile eHL preference, and knowledge and skills of computers, internet, and mobile technology (n=249).

Scale		Mean	SD	Items	Range	Min	Max	Reliability (α)
**Moblie eHL**								
	eHL	30.16	5.41	8	8-40	8	40	.927
	mHL	28.86	6.27	8	8-40	8	40	.927
	Mobile eHL preference	14.65	2.57	4	4-20	8	20	.847
**Knowledge and skills^a^**								
	Knowledge	13.96	2.66	15	0-15	0	15	.928
	Skills	8.54	2.74	10	0-10	0	10	.923
Self-rated health		6.70	1.77	3	0-3	0	3	.546
Self-care behavior		79.30	26.05	36	0-144	17	144	.935

^a^Kuder-Richardson Formula 20 (KR-20) reliability for knowledge and skills of computers, the internet, and mobile technology.

### Association and Exploratory Structural Equation Modeling Analyses

The bivariate relationships among the variables are shown in Multimedia Appendix 3. The 3 subscales of mobile eHL are moderately to highly correlated (eHL and mHL, *r*=0.764, *P<*.001; eHL and mobile eHL preference, *r*=0.577, *P<*.001; mHL and mobile eHL preference*, r*=0.515, *P*<.001). The correlations between mobile eHL and self-care behavior are significant (eHL, *r*=0.157, *P*=.013; mHL, *r*=0.188, *P*=.003; mobile eHL preference*, r*=0.211, *P*<.001). Both knowledge and skills of computers, the internet, and mobile technology are also significantly correlated with mobile eHL (*rho*=0.231 to 0.466, *P*<.001). However, the eHL and mHL literacy scores have significant and negative associations with age (eHL, *r*=-0.380, *P*<.001; mHL, *r*=-0.398, *P=*.036) and diabetes duration (eHL, *r*=-0.159, *P*=.013; mHL, *r*=-0.135, *P*<.001). The self-care behavior is reversely correlated with hours of smartphone daily use (*r*=-0.139, *P=*.033) but not related to HbA_1c_ or self-rated health. Participants with many hours of smartphone use may have poor self-care behavior. Self-rated health is significantly and negatively associated with HbA_1c_ (*r*=-0.290, *P*<.001). In turn, the results revealed that a higher level of self-rated health was associated with a lower HbA_1c_ level.

**Figure 1:**
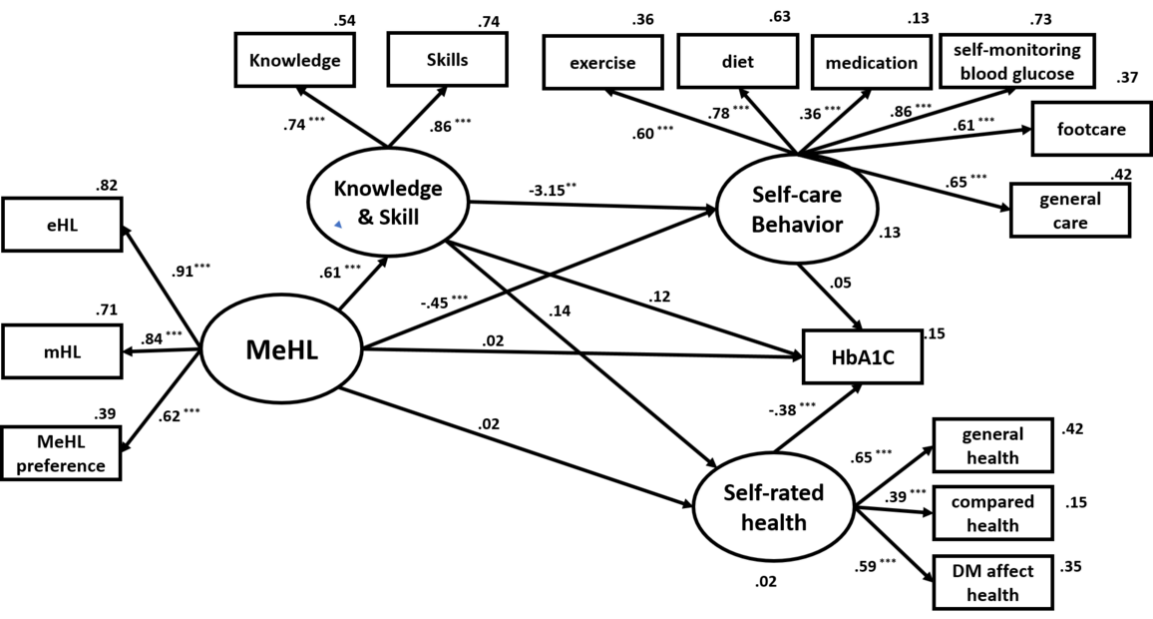
The mobile eHealth literacy (MeHL) model that was first tested. DM: diabetes mellitus; eHL: eHealth literacy; HbA_1c_: glycated hemoglobin; mHL: mobile health literacy.*P=.01-.05, **P=.001-0.01<.01, ***P<.001.

**Figure 2:**
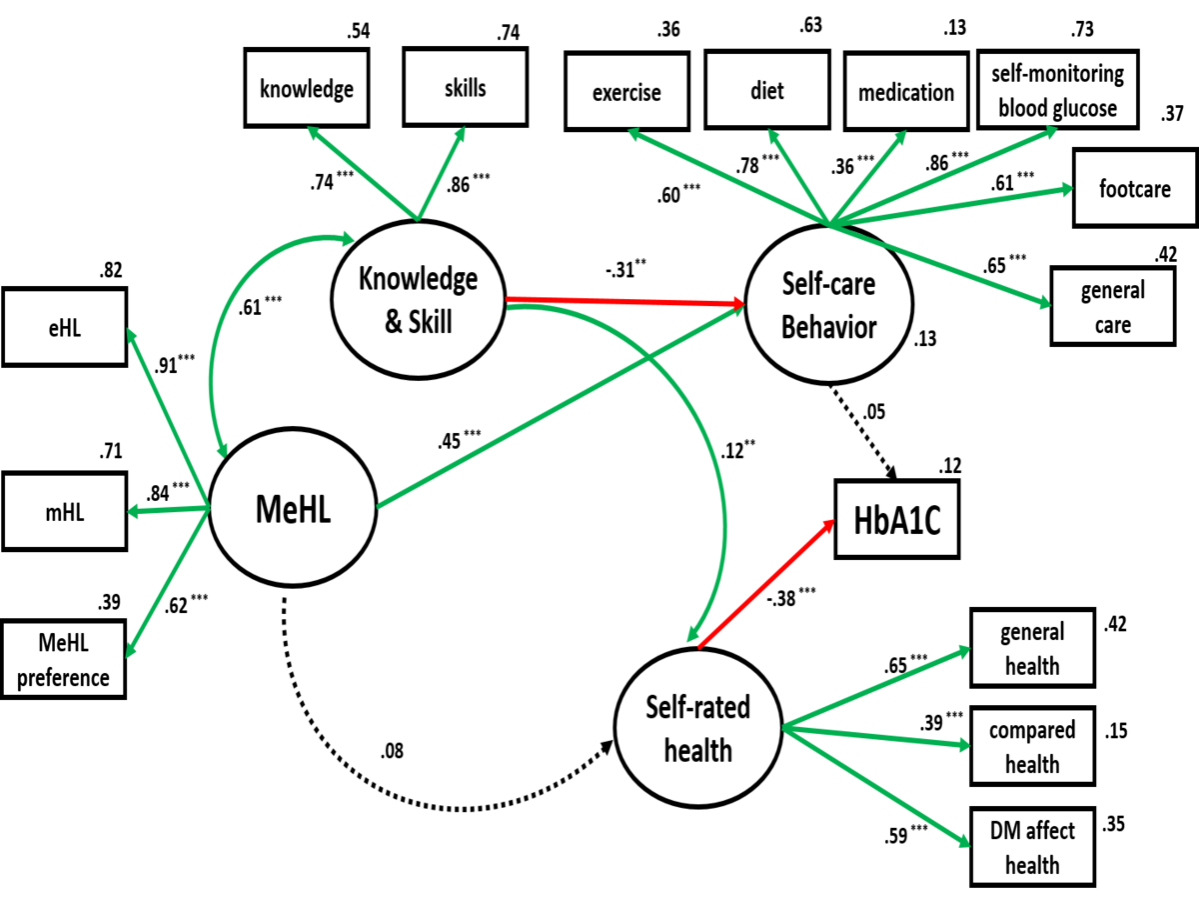
A pruned model of mobile eHealth literacy (MeHL), self-care behavior, and glycemic control. DM: diabetes mellitus; eHL: eHealth literacy; HbA_1c_: glycated hemoglobin; mHL: mobile health literacy. *P=.01-.05, **P=.001-0.01<.01, ***P<.001.

An SEM approach was used to explore the structural relationships among the variables. Mobile eHealth literacy, a dependent latent variable, was evaluated through independent latent variables, including eHL, mHL, and mobile eHL preference. The initial structural model is shown in Figure 1. The first model was overly complex and did not achieve a satisfactory fit. Figure 2 presents the pruned model in which some paths were changed. Moreover, a relationship based on mutual influence was proposed between knowledge and skills, and mobile eHL. The refined model had adequate goodness-of-fit indexes: χ^2^_83_=149.572, *P*<.001; comparative fit index (CFI)=0.925; root mean square of approximation (RMSEA)=0.057 (90% CI 004-006); χ^2^/*df* = 1.082. The mobile eHL had a weak indirect effect on self-rated health through the variables of knowledge and skills. The self-rated health score exerted a significant direct effect on HbA_1c_. However, the result reveals a nonsignificant direct influence on the mobile eHL at HbA_1c_ and self-rated health, and lower-than-expected coefficients for this domain's pathways.

## Discussion

### Principal Findings

Firstly, this study conducted a survey that measures mobile eHL and the use of health information technology. Most of the respondents had searched for online diabetes information previously, but only a few respondents had used health apps. Some demographic factors affecting mobile eHL included age, education, and duration of type 2 diabetes. Secondly, this study further examined the link between mobile eHL and health outcomes. Mobile eHL is related to self-care behaviors as well as knowledge and skills of computers, internet, and web mobile technology, but only has a weak, indirect effect on self-rated health. This highlights the value of mobile eHL as a crucial indicator of the ability to embrace health information technology. Finally, the participants with higher mobile eHL were more likely to achieve effective self-care behavior.

### The Use of eHealth and mHealth Technology

The respondents reported using their smartphones for an average of 6.5 years, with an average daily use of 4.5 hours. Consistent with previous survey findings [5,28], our findings indicated that over 60% of the participants had looked for diabetes-related information on the internet, but only a very small portion had previously used health apps. Participant education level was high, with 60% of participants having at least a degree from college or university; participants also reported moderate confidence in using mobile eHealth technology. However, a comparison between smartphone use (96%) and mHealth app adoption (1.6%) seems to have a rather large discrepancy in this study. In other words, the mobile eHL results appear to be out of sync with their actual experience. It is possible that the participants were unfamiliar with current health apps. A national survey by Zhang et al [28] found that the awareness rate of diabetes apps was 29.94%, and usage was 15.44%. Another study also noted that many patients were unaware of mHealth apps [18]. These findings suggest that future diabetic populations should be made aware of available mHealth apps.

As expected, the results show that the participants with higher education had a greater level of mobile eHealth literacy, but the duration of type 2 diabetes had an inverse relationship with mobile eHealth literacy. These findings are also consistent with James and Harville's report [5]. Previous studies found [11,18,44] that patients experienced basic computer obstacles and perceived barriers in the use of diabetes apps. To further identify these potential hurdles, the mobile eHL measure proposed in this study can be used as an additional screening tool, serving as a strong proxy for identifying patients who need the most support in using health information and mHealth apps.

Above all, people with diabetes may need a clear reference for which apps to download and use; this reference can come from peers with diabetes and health care providers. Although most people use mobile apps daily, this does not necessarily mean that they would be able to employ an mHealth app for handling diabetes. For example, inputting blood glucose data in most diabetes apps requires one to complete several steps, including essential registration involving an email address, personal body weight, height, social security number, password, etc. These digital access and literacy disparity problems might diminish if clinical practitioners were responsible for mobile eHealth education. Also, several approaches might promote mHealth app engagement going forward; for example, social networking app strategies (eg, WhatsApp, Line, or WeChat) can be applied to mHealth apps to increase their popularity and effectiveness. Furthermore, researchers may consider sociodemographic factors such as education and age, which may differently influence people's acceptance of mobile eHealth technology.

### SEM Analysis of the Relationship Between Mobile eHealth Literacy and Patient Outcomes

Testing of the hypotheses revealed a positive relationship between mobile eHealth literacy and self-care behavior. Our results are not directly comparable to previous studies on eHL models [16,24,25] since their results did not include mHealth app use literacy. Measures of mHL are relatively few, and its evidence is accumulating in this field.

Hypothesis 1, which states that higher mobile eHL is positively associated with knowledge and skills and increased self-care behaviors, was supported. The result demonstrated that higher mobile eHL only had an indirect connection to better self-rated health status. Similarly, Schulz et al [25] found that eHL was directly related to health information-seeking behavior and only indirectly related to health care system utilization. Critically, the interlocking relationship between mobile eHealth literacy and self-care behaviors can serve to disclose self-care practices. In short, mobile eHL may shape the behavioral responses of the diabetes population.

In more statistical terms, higher mobile eHL cannot be assumed to reduce HbA_1c_ in this study. The research linking eHealth literacy to health outcomes based on cross-sectional data [25,31,45] has mixed results. Another similar concept, health literacy, has neither a direct nor indirect effect on health outcomes (diabetes knowledge and glycemic control); it only has an indirect effect through its association with social support [27]. A recent study of chronic disease indicated that eHealth literacy was correlated to disease-related self-care behaviors [45]. A longitudinal design will provide an opportunity to determine the need to yield more evidence in such a situation.

Hypothesis 2 is just the opposite. Knowledge and skills of computers, the internet, and mobile technology was negatively related to self-care behaviors. Multimedia Appendix 3 shows that spending more time with smartphones was significantly related to poor self-care behaviors. Unfortunately, there are no similar studies to compare with the unexpected result that people with diabetes with higher knowledge and skills about technology will tend to exhibit fewer self-care behaviors. One possible explanation is that those who have higher skills and knowledge of computers, the internet, and web mobile technology may be more immersed in outward-looking technology, leading them, in turn, to pay less attention to self-care activities. Further studies are needed to address this possibility.

Unsurprisingly, the relationship between self-rated health status and glycemic control indicates that higher perceived health status was related to lower HbA_1c_. However, HbA_1c_ cannot be predicted by self-care behaviors. Our results did not agree with the previous behavior-to-outcomes study [32]. The connections between psychodynamic variables and behavioral variables are complex, perhaps because HbA_1c_ is a 3-month average of glycemic levels, which is a much longer time frame than the other measured variables. More research is needed to elucidate the underlying factors between relationship models, such as adding time considerations.

The bivariate correlation results show more significant relationships than the results of the SEM model. Our results are in line with scholars [46] who have reported a difference between the correlation coefficient and the path coefficient of the SEM model. According to the tracing rule [46], the correlation between any pair of variables equals the sum of the products of the paths or correlations from each tracing. Thus, the significance levels of the variables in our hierarchical model are diminished when weak or negative relationships exist. Benitez et al [47] concluded that it remains indispensable to assess all path coefficients and their significance regardless of whether one performs confirmatory or explanatory research.

### Limitations

A possible limitation of this study is that the adult subjects are from the outpatient departments of hospitals in Taiwan. Therefore, the study findings may not be generalized to other populations with diabetes. Other limitations include self-reports and a cross-sectional design, the latter precluding inference on causality. Thirdly, the selection of the participants was not random. Finally, this study did not analyze information on other factors believed to explain the relationship between mobile eHealth literacy and health outcomes in the SEM model.

Despite the above limitations, this study provides some meaningful results. Whereas some paths do not show the correlation between these variables, they do have an impact on the overall model. Many internet use aspects were examined, providing new information about the experiences, opinions, and attitudes of people with diabetes toward computers and the internet. In addition, for the first time, the factors of mobile eHealth literacy among people with diabetes were described and compared. This provides valuable insights into the eHealth literacy and the mHealth app experience of a population with diabetes.

Smart health technology can improve disease conditions. Assessing mobile eHealth literacy can help identify the gaps in knowledge and skills among patients with diabetes. Most patients with diabetes were less likely to use mHealth apps, and their mobile eHealth literacy was found to be moderate. This is a large group that can potentially use diabetes apps after enhancing their literacy. Their eHealth literacy was found to be moderate. Further research should identify more variables that mediate the relationship between mobile eHealth literacy and health care outcomes.

### Implications

This study's main contribution to the body of knowledge is its enhancement of the mobile eHealth literacy model to understand the relationships of diabetes outcomes. The study highlights the importance of health professionals' awareness of this literacy so that they can appropriately tailor their interventions with patients. Providers can also serve as an educational source of support to enhance patients' health-related internet use abilities and to match suitable health information from the internet to the needs of patients with chronic conditions. The value of using health information technology to provide information for chronic disease self-management may be limited if it does not include support from health care providers.

The mobile mHealth literacy survey tool should be integrated into chronic care delivery, enabling health care providers to measure diabetes technology implementation. This knowledge will help to create a fuller conception of an analytical framework of mobile eHealth literacy.

## Appendix

Multimedia Appendix 1 CONSORT-eHEALTH V1.6.1.

Multimedia Appendix 2 Mobile eHealth literacy questionnaire.

Multimedia Appendix 3 Correlation between mobile eHealth literacy (MeHL) variables and patients’ outcomes (n=249). eHL: eHealth literacy; HbA_1c_: glycated hemoglobin; mHL: mobile health literacy.

## Abbreviations

CFI: comparative fit index
eHL: electronic health literacy
HbA_1c_: glycated hemoglobin
mHL: mobile health literacy
mobile eHL: electronic health literacy and mobile health literacy
RMSEA: root mean square of approximation
SEM: structural equation model
