# Supplementary material for: Relationships Between Mobile eHealth Literacy, Diabetes Self-care, and Glycemic Outcomes in Taiwanese Patients With Type 2 Diabetes: Cross-sectional Study
Source: JMIR Mhealth Uhealth. 2021 Feb 5;9(2):e18404. doi: 10.2196/18404 (PMC7895642; doi:10.2196/18404)
Supplement: Multimedia Appendix 2 [file mhealth_v9i2e18404_app2.docx]

**Multimedia Appendix 2.** Mobile eHealth literacy questionnaire.

| Items |
| --- |
|  |
| **eHealth literacy** *(Strongly Disagree to Strongly Agree)* |
| 1. I know what health resources are available on the Internet |
| 1. I know where to find helpful health resources on the Internet |
| 1. I know how to find helpful health resources on the Internet |
| 1. I know how to use the Internet to answer my questions about health |
| 1. I know how to use the health information I find on the Internet to help me |
| 1. I have the skills I need to evaluate the health resources I find on the Internet |
| 1. I can tell high quality health resources from low quality health resources on the Internet |
| 1. I feel confident in using information from the Internet to make health decisions |
| **mHealth Literacy** *(Strongly Disagree to Strongly Agree)* |
| 1. I am familiar with operation of smart devices applications, such as line app, game apps |
| 1. I know how to download health apps on smart devices |
| 1. I feel confident in using health apps on smart devices |
| 1. I know how to use diabetes apps to record my health data (such as blood sugar) |
| 1. I know how to use diabetes apps to help me manage my disease (such as medication reminder) |
| 1. I can appraisal which apps is proper to myself condition |
| 1. I can tell the quality of health apps (quality means: the functionality and content of apps |
| 1. I feel confident in using diabetes apps to manage my health status |
| **Mobile eHealth Preference** |
| 1. How useful do you feel *the Internet* is in helping you in making decisions about your health? *(Answer options: Not useful at all…. Very Useful)* |
| 1. How important is it for you to be able to access *health resources on the Internet*? *(Answer options: Not important at all…. Very Important)* |
| 1. How useful do you feel *Health Ap*ps is in helping you in making decisions about your health? *(Answer options: Not useful at all…. Very Useful)* |
| 1. How important is it for you to be able to access *Health Ap*ps? *(Answer options: Not important at all…. Very Important)* |
