# Supplementary material for: Relationships Between Mobile eHealth Literacy, Diabetes Self-care, and Glycemic Outcomes in Taiwanese Patients With Type 2 Diabetes: Cross-sectional Study
Source: JMIR Mhealth Uhealth. 2021 Feb 5;9(2):e18404. doi: 10.2196/18404 (PMC7895642; doi:10.2196/18404)
Supplement: Multimedia Appendix 3 [file mhealth_v9i2e18404_app3.docx]

**Multimedia Appendix 3.** Correlation between mobile eHealth literacy (MeHL) variables and patients’ outcomes (n=249). eHL: eHealth literacy; HbA1c: glycated hemoglobin; mHL: mobile health literacy.

| Scales | 1 | 2 | 3 | 4 | 5 | 6 | 7 | 8 | 9 | 10 | 11 | 12 | 13 |
| --- | --- | --- | --- | --- | --- | --- | --- | --- | --- | --- | --- | --- | --- |
| 1 Age ^a^ | — |  |  |  |  |  |  |  |  |  |  |  |  |
| 2 Education ^b^ | -.248 (<.001) | — |  |  |  |  |  |  |  |  |  |  |  |
| 3 Duration of diabetes ^a^ | .268  (<.001) | -.072  (.260) | — |  |  |  |  |  |  |  |  |  |  |
| 4 Daily use of smart phone ^a^ | -.214  (0.001) | .119  (.070) | -.135  (0.041) | — |  |  |  |  |  |  |  |  |  |
| 5 Daily use of computer and Internet ^a^ | -.162  (.168) | .267  (.001) | .020  (.806) | .298  (<.001) | — |  |  |  |  |  |  |  |  |
| 6 eHL ^a^ | -.380  (<.001) | .340  (<.001) | -.159  (.013) | .126  (.053) | -.108  (.173) | — |  |  |  |  |  |  |  |
| 7 mHL ^a^ | -.398  (<.001) | .294  (<.001) | -.135  (0.036) | .141  (.031) | .036  (.647) | .764  (<.001) | — |  |  |  |  |  |  |
| 8 MeHL preference ^a^ | -.176  (.006) | .191  (.003) | -.092  (.152) | .124  (.059) | .058  (.472) | .577  (<.001) | .515  (<.001) | — |  |  |  |  |  |
| 9 Knowledge ^a^ | -.379  (<.001) | .378  (<.001) | -.193  (.002) | .224  (.001) | .141  (.075) | .437  (<.001) | .395  (<.001) | .231  (<.001) | — |  |  |  |  |
| 10 Skills ^a^ | -.473  (<.001) | .419  (<.001) | -.197  (.002) | .261  (<.001) | .119  (.133) | .466  (<.001) | .458  (<.001) | .287  (<.001) | .631  (<.001) | — |  |  |  |
| 11 Self-rated health ^a^ | .283  (<.001) | .005  (.942) | -.090  (.161) | .023  (.723) | -.011  (.894) | -0.004  (.945) | -.026  (.679) | .050  (.436) | 0.048  (.446) | 0.026  (.683) | — |  |  |
| 12 self-care Behavior ^a^ | -.026  (.678) | .006  (.924) | .126  (.049) | -.139  (.033) | -.081  (.304) | .157  (.013) | .188  (.003) | .211  (<.001) | -0.007  (.917) | -0.082  (.196) | .113  (.075) | — |  |
| 13 HbA1C ^a^ | -0.242  (<.001) | -.160  (.012) | .064  (.322) | .069  (.290) | .007  (.926) | .074  (.243) | .046  (.474) | -0.019  (.762) | .008  (.894) | .083  (.191) | -.290  (<.001) | 0.02  (.756) | — |
| ^a^ shown as Pearson correlation coefficient r (P value); ^b^ shown as Spearman correlation coefficient r (P value)  Skill = skill about computer/web/mobile; Knowledge = knowledge about computer/web/mobile | | | | | | | | | | | | | |
